# Carcinoma of the colon

**DOI:** 10.1054/bjoc.2001.1767

**Published:** 2001-05

**Authors:** A Adenis, T Conroy, P Lasser, Y Merrouche, G Monges, M Rivoire, P Rougier, S Ruggieri-Pignon

**Affiliations:** Centre Oscar Lambret, Lille; Centre Alexis Vautrin, Nancy; Institut Gustave Roussy, Villejuif; CHRU, Besançon; Institut Paoli-Calmettes, Marseille; Centre Léon Bérard, Lyon; Hôpital Ambroise Paré, Boulogne, France


British Journal of Cancer (2001) **84** (Supplement 2), 65–68
© 2001 FNCLCC
doi: 10.1054/ bjoc.2001.1767, available online at http://www.idealibrary.com on IDEAL®

### Carcinoma of the colon

**A Adenis[1], T Conroy[2], P Lasser[3], Y Merrouche[4], G Monges[5], M Rivoire[6], P Rougier[7] and S Ruggieri-Pignon[5]**

[1]Centre Oscar Lambret, Lille; [2]Centre Alexis Vautrin, Nancy; [3]Institut Gustave Roussy, Villejuif; [4]CHRU, Besançon; [5]Institut Paoli-Calmettes, Marseille; [6]Centre Léon Bérard, Lyon; [7]Hôpital Ambroise Paré, Boulogne, France

Cancer of the colon has an estimated incidence of 31 per 1000 per year and causes the death of 18.5 per 1000. Adenocarcinoma of the colon represents 60% of all colorectal cancers diagnosed in France.

This document refers to the management of adenocarcinomas of the colon which account for 90–95% of all large bowel tumours. It does not consider the more rare tumours such as neuroendocrine tumours, sarcomas or melanomas.

These guidelines were validated in April 2000 by the working group and an update is planned for 2001.

### DIAGNOSIS AND PREOPERATIVE STAGING

Colonoscopy and biopsy is the only way to make a definitive diagnosis of cancer of the colon (standard). A barium enema can be used in cases where colonoscopy is difficult (for example if the tumour cannot be reached with the colonoscope), to localize a small tumour, or to search for synchronous lesions (option).

Preoperative staging must include a complete clinical examination, appropriate blood tests and an enquiry into the family history. Chest X-ray and abdominal ultrasound are used for the assessment of metastatic spread (standard). Analysis of the level of carcino-embryonic antigen (CAE) is optional; it is has not been clearly demonstrated that this test affects the therapeutic plan. An abdominal CT scan is useful in the search for metastatic disease in patients who are obese, if serial imaging is necessary to follow the disease during treatment and for patients in whom ultrasonography is difficult.

### CLASSIFICATION

The standard post-therapeutic classifications (the UICC-AJCC, Astler-Coller and Dukes classifications) all facilitate subsequent treatment decisions. The classifications of Gunderson and Sosin and of the GITSG are options.

### PROGNOSTIC FACTORS

The standard prognostic factors are the extent of bowel wall infiltration, contiguous invasion of adjacent organs, nodal involvement, the number of nodes involved, the number of nodes removed and the presence of metastases at the time of preoperative staging. Further studies are necessary to identify or confirm the value of other prognostic factors.

### TREATMENT MODALITIES

### Surgery

#### *Preparation for surgery*

The standard bowel preparation includes a thorough bowel washout with a hypertonic solution combined with a low-residue diet, an intravenous injection of a broad-spectrum antibiotic and the marking of planned stoma sites.

#### *Routine surgical treatment of cancer of the colon*

This comprises the excision of the primary tumour with safe margins and the excision of vessels and associated mesocolon containing lymphatic channels and nodes. A median laparotomy incision is recommended, followed by an examination of the liver, pelvis and ovaries (in women), with sampling or frozen section of each suspicious mass. Several procedures can be undertaken depending on the site of the tumour: hemicolectomy (right or left), resection of the transverse colon, sigmoidectomy or an anterior resection of the rectosigmoid.

#### *Surgical treatment of complex tumours*

There is no standard approach to the surgical treatment of tumours presenting with bowel obstruction or perforation. It will depend on the performance status of the patient and the location of the tumour. If the tumour has invaded neighbouring organs, the resection should be made ‘en bloc’ (standard).

A subtotal colectomy is recommended for the treatment of Lynch syndrome.

In post-menopausal women, prophylactic bilateral oophorectomy is recommended. Palliative resection of a colon cancer is an option, depending on the performance status of the patient, the ease of excision and whether or not the patient is symptomatic.

Laporoscopic colectomy must not be undertaken outside a therapeutic trial.

#### *Bowel anastomosis*

An effective anastomosis requires good bowel preparation. The vascular supply to adjacent bowel segments must be well maintained and should not be subject to undue traction (standard). Mechanical and manual techniques for anastomosis (staples vs stitches) give the same results in the hands of experienced surgeons. In the case of a manual anastomosis, a one-layer anastomosis is recommended.

#### *Hepatic metastases*

The operability criteria are anatomic (surgery is contra-indicated if the portal and/or sub-hepatic veins are involved) and technical (the necessity of leaving more than 30% of normal liver 'in situ'). It will also depend on the extent of disease spread (whether or not there are nodes at the porta hepatis, the presence of non-resectable metastases outside the liver, the number and size of metastases, etc) (level of evidence B). Surgery for the excision of hepatic metastases must result in the removal of all metastases demonstrated by extensive preoperative scanning. If possible, each metastasis must be surrounded by a margin of 1 cm of healthy liver (level of evidence C). The aim is to preserve as much normal tissue as possible while limiting the amount of blood loss utilizing hepatic vascular exclusion. The hepatectomy technique is based on anatomic hepatic segments and depends on the size, the number and position of the metastases. There is no evidence that hepatectomy gives better results than metastectomy (level of evidence B).

### Radiotherapy

The role of radiotherapy in the treatment of cancer of the colon has not been clearly defined. It is an option for the treatment of incompletely excised tumours, especially when the residual tumour is small and when no other potentially curative therapy is planned.

### Chemotherapy

#### *Adjuvant chemotherapy*

For stage III colon cancer, adjuvant chemotherapy is standard (level of evidence A). A 6-month course of combination of 5-fluorouracil (5-FU) and folinic acid (FA) is standard (level of evidence A). The early results of therapeutic trials of post-operative intraportal chemotherapy are conflicting (level of evidence C).

For stage I colon cancers there is no indication for adjuvant treatment (standard, level of evidence B). The use of adjuvant chemotherapy for stage II disease remains experimental.

#### *Palliative chemotherapy*

Chemotherapy is recommended for the palliative treatment of inoperable cancers of the colon (level of evidence B). Two combinations, 5-FU/FA and 5-FU/methotrexate ± FA (5-FU-MTX ± FA) are superior in terms of response and toxicity than 5-FU alone administered by intravenous bolus (level of evidence A). Only 5-FU-MTX ± FA gives a statistically significant increase in median survival. In a randomized study, the LV 5-FU2 protocol has been shown to be more effective (in terms of response and progression-free survival) and less toxic than the Mayo Clinic 5-FU/FA combination, but without a statistically significant increase in survival (level of evidence B).

Continuous infusions of intravenous 5-FU (level of evidence B) or of raltitrexed (level of evidence C) are options having comparable efficacy to the 5-FU/FA combination.

The addition of oxaliplatin to a 5-FU/FA combination increases the toxicity and the response rate. The median progression-free survival is increased by nearly 3 months using oxaliplatin with no difference in median survival (level of evidence B). The addition of irinotecan to a 5-FU/FA combination also increases the toxicity and the response rate, with a 2-month delay in disease progression and a significant 3-month improvement in overall survival. There is no difference in quality of life between the two groups (level of evidence B). The combination of oxaliplatin or irinotecan with 5-FU/FA as first-line treatment for metastatic disease is optional (level of evidence B). The value of a triple chronomodulated infusion of oxaliplatin, 5-FU and FA has not been established in that it has not been compared with a standard cytotoxic regimen.

Hepatic intra-arterial chemotherapy with floxuridine (FUDR) is an option (level of evidence B) for the treatment of inoperable hepatic metastases. Its value compared to best systemic treatment has not been established.

### Immunotherapy

The efficacy of non-specific immunotherapy and of vaccines has not been proven. The place of monoclonal antibodies remains to be confirmed; they should only be used within the framework of therapeutic trials (option, level of evidence C).

### The management of polyps

A decision regarding surgery in the management of polyps must be made on a case-by-case basis by the gastroenterologist, pathologist, surgeon and physician caring for the patient.

Strict follow-up is necessary after polypectomy. The risk of subsequently developing a cancer is much greater if the polyp is villous or tubulovillous and greater than 1 cm in size. For patients with a non-invasive pedicular or sessile adenomatous polyp of less than 3 cm in size at the base that has been completely resected along with any other polyps, a colonoscopy must be performed 3 years after the initial excision. For all other patients, colonoscopy must be undertaken within a year of the initial excision. Thereafter, routine surveillance of bowel lesions can be instituted.

### THERAPEUTIC STRATEGY

Surgery is the main treatment for colon cancer, with the addition of complimentary or adjuvant treatment as appropriate.

### Adjuvant treatment after curative surgery

**i)** There is no indication for additional treatment before obtaining a complete histological report (standard). Immediate postoperative chemotherapy with intraportal 5-FU can be given within a therapeutic trial (option, level of evidence C) (Figure 1).

**ii)** After obtaining a complete histological report:

#### *Stage I T1 N0 M0 (Dukes A, Astler-Coller A) or T2 N0 M0 (Dukes A, Astler-Coller B1) disease*

There is no indication for adjuvant treatment following resection (standard)

#### *Stage II T3 or T4 N0 M0 (Dukes B, Astler-Coller B2)*

There is no indication for adjuvant treatment following resection (standard). The inclusion of patients within therapeutic trials is recommended.

#### *Stage III, any T, NI–3, M0 (Dukes C, Astler-Coller C1 and C2)*

Adjuvant systemic chemotherapy is the standard treatment (level of evidence A). The combination of 5-FU and FA is standard (level of evidence A)

#### *Colon cancer presenting with bowel perforation or obstruction*

There is no standard therapy. Adjuvant systemic chemotherapy or the inclusion in a therapeutic trial are options.

 *© 2001 FNCLCC*

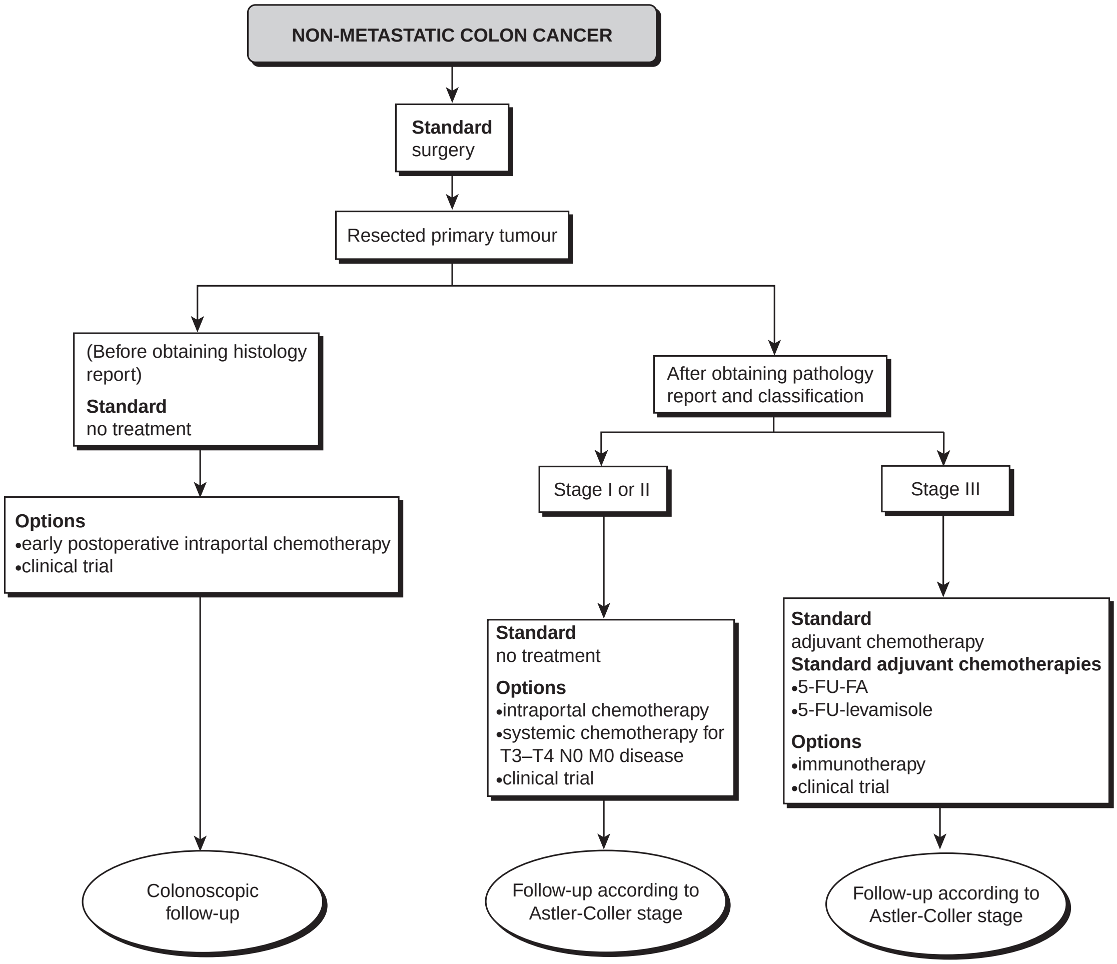

**Figure 1** Treatment of resectable colon cancer

### Treatment of non-resectable metastatic colon cancer

In metastatic disease, the aim of chemotherapy based on 5-FU is to improve the duration of survival with maintained or improved quality of life (Figures 2 and 3).

It should be given before the appearance of symptoms to patients with a performance status of 0 1 or 2 who have been fully informed of the implications of treatment and its side-effects, who have proven pathology and unresectable metastatic disease. Standard chemotherapy is a 5-FU-based protocol of low toxicity (level of evidence B).

The efficacy of any palliative chemotherapy protocol must be evaluated after 2–3 months' treatment. There is no standard for treatment following a good response to chemotherapy. Surgery (if possible), or further chemotherapy are options. Chemotherapy must be discontinued in the face of progressive disease (standard). Second-line chemotherapy using a different protocol (level of evidence B) or the inclusion in a therapeutic trial are then options.

### FOLLOW-UP

The purpose of follow-up after potentially curative surgery for carcinoma of the colon is to identify a recurrence as early as possible. The benefit of this is uncertain. No randomized prospective study has shown an increase in survival or quality of life following a strategy of surveillance. Follow-up should include a clinical examination (standard) followed by endoscopy/colonoscopy (standard).

Colonoscopy must be undertaken within 12 months of surgery (6 months if a preoperative colonoscopy was incomplete or not done) and repeated according to the findings (standard). For Stage II and III disease (Astler-Coller stage B2 and C), follow-up can be

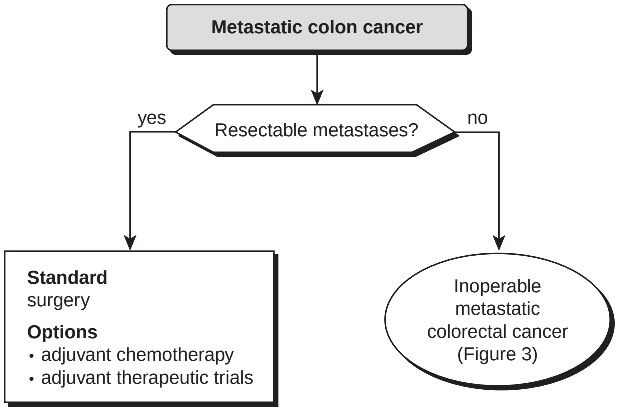

**Figure 2** Treatment of metastatic colon cancer

© 2001 FNCLCC

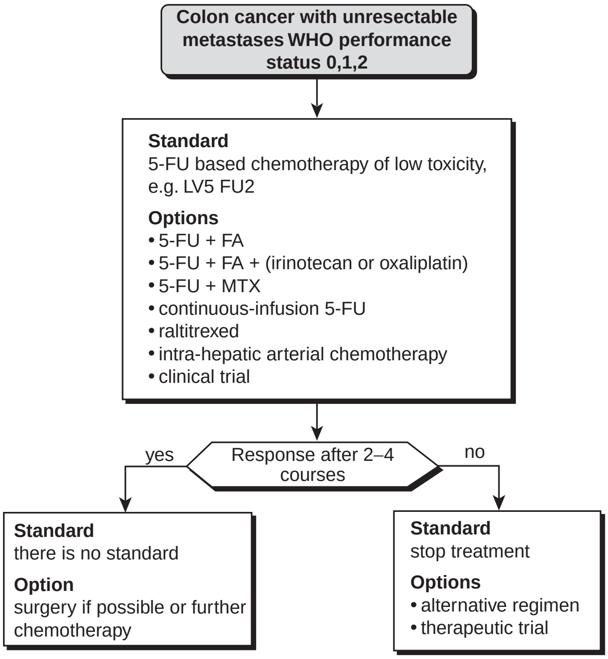

**Figure 3** Treatment of metastatic colon cancer with unresectable metastases intensified to identify those patients who are potentially re-operable and combined with an analysis of the level of CEA (option).

Appropriate imaging includes liver ultrasonography (for example every 3 months for 2 years, then every 6 months for 5 years) plus an annual chest X-ray (option). Prospective randomized studies must be carried out to evaluate the benefit of follow-up and to define the best methods, frequency and cost-effectiveness.

### INTERNAL REVIEWERS

Y Bécouarn (Institut Bergonié, Bordeaux), C Borel (Centre Paul Strauss, Strasbourg), A Boudinet (Centre René Huguenin, Saint-Cloud), R Brunet (Institut Bergonié, Bordeaux), G Depadt (Centre Oscar Lambret, Lille), JY Doullard (Centre René Gauducheau, Nantes), D de Raucourt (Centre François Baclesse, Caen), M Ducreux (Institut Gustave Roussy, Villejuif), D Elias (Institut Gustave Roussy, Villejuif), J Fraisse (Centre Georges-François Leclerc, Dijon), E François (Centre Paul Strauss, Strasbourg), JH Jacob (Centre François Baclesse, Caen), G Lorimier (Centre Paul Papin, Angers), J Mihura (Centre Claudius Regaud, Toulouse), JC Ollier (Centre Paul Strauss, Strasbourg), B Paillot (Centre Henri Becquerel, Rouen), P Pouillart (Institut Curie, Paris), JL Raoul (Centre Eugène Marquis, Rennes), B Saint-Aubert (Centre Val d'Aurelle, Montpellier), C Schumacher (Centre Paul Strauss, Strasbourg), JF Seitz (Institut Paoli Calmettes, Marseille), P Troufléau (Centre Alexis Vautrin, Vandœuvre-Lès-Nancy) and P Wagner (Centre Paul Strauss, Strasbourg).

### EXTERNAL REVIEWERS

JM Ardiet (Clinique Saint-Jean, Lyon), P Ardisson (Lyon), JP Arnaud (CHU, Angers), N Barbet (Macon), L Bedenne (CHU, Dijon), J Belfort (Le Mans), C Berger (Clinique Sainte-Catherine, Avignon), H Bleiberg (Institut Jules Bordet, Bruxelles – Belgique), P Boissel (CHU Brabois, Vandœuvre-Lès-Nancy), JF Bosset (Hôpital J Minjoz, Besancon), PM Bret (The Montreal General Hospital, Montreal – Canada), P Burtin (CHU Angers, Angers), E Calitchi (Boulogne), L Cany (Périgueux), P Colin (Courlancy, Nancy), T de Dombal (Clinical Information Science unit, University of Leeds, Leeds – Angleterre), A de Gramont (Hôpital Saint-Antoine, Paris), A Deleuze (Ales), C de Seguin (Polyclinique, Ganges), B Detroz (CHU, Liège), JP Dujols (Centre de Radiothérapie et d'Oncologie médicale, Pau), JP Dutin (Centre Saint-Michel, La Rochelle), F Economides (Dunkerque), PL Etienne (Clinique Armoricaine de Radiologie, Saint-Brieuc), J Faivre (CHU, Dijon), J Fournet (CHU, Grenoble), D Fric (Clinique du Mail, Grenoble), G Ganem (Centre Jean Bernard, Le Mans), C Georgeac (Clinique Saint-Côme, Le Mans), M Gignoux (Hôpital Côte de Nacre, Caen), S Greget (Clinique Sainte-Clotilde, Sainte-Clotilde), F Guichard (Bordeaux), P Kitmacher (La Rochelle), H Labrosse (Cabinet « La Giraldière », Lyon), R Lambert (CHU Edouard Herriot, Lyon), D Langlois (La Rochelle), P Lucas (Polyclinique Les Bleuets, Reims), P Martin (Clinique Saint-Jean, Lyon), M Marty (Hôpital Saint-Louis, Paris), O Maton (Clinique de Francheville, Perigueux), M Moreau, Centre hospitalier (Gleize), M Moro (Nice), B Nordlinger (Hôpital Ambroise Paré, Boulogne), H Orfeuvre (Centre Hospitalier, Bourg-en-Bresse), R Otter (Integraal Kankercentrum Noordnederland, Gronigen – Pays Bas), E Pelissier (Centre médical Château Galland, Besançon), M Piccard (Institut J Bordet, Bruxelles – Belgique), P Piedbois (Hôpital Henri Mondor, Créteil), P Pienkowski (Clinique Pont de Chaume, Montauban), T Ponchon (CHU-Edouard Herriot, Lyon), X Pouliquen (Centre Hospitalier Victor Dupouy, Argenteuil), F Reboul (Clinique Sainte-Catherine, Avignon), P Revelin (Centre Hospitalier, Roanne), I Rossions (Deutsche Krebsgesellschaft Ev, Francfort – Allemagne), R Soleilhac (Clinique Sainte-Clotilde, Sainte-Clotilde), E Tiret (Hôpital Saint-Antoine, Paris) and B Watrin (Clinique d'Essey, Essey-Lès-Nancy).

*© 2001 FNCLCC*

